# Evaluation of the Environmental Performance of Adsorbent Materials Prepared from Agave Bagasse for Water Remediation: Solid Waste Management Proposal of the Tequila Industry

**DOI:** 10.3390/ma16010008

**Published:** 2022-12-20

**Authors:** Camila S. Gómez-Navarro, Walter M. Warren-Vega, Juan C. Serna-Carrizales, Ana I. Zárate-Guzmán, Raúl Ocampo-Pérez, Francisco Carrasco-Marín, Virginia H. Collins-Martínez, Joaquina Niembro-García, Luis A. Romero-Cano

**Affiliations:** 1 Grupo de Investigación en Materiales y Fenómenos de Superficie, Departamento de Biotecnológicas y Ambientales, Universidad Autónoma de Guadalajara, Av. Patria 1201, Zapopan 45129, Mexico; 2 Centro de Investigación y Estudios de Posgrado, Facultad de Ciencias Químicas, Universidad Autónoma de San Luis Potosí, San Luis Potosí 78260, Mexico; 3 Grupo de Investigación en Materiales de Carbón, Facultad de Ciencias, Universidad de Granada, Av. Fuente Nueva s/n, 18071 Granada, Spain; 4 Centro de Investigación en Materiales Avanzados (CIMAV), S.C. Miguel de Cervantes #120, Complejo Industrial Chihuahua, Chihuahua 31136, Mexico; 5 Facultad de Ingeniería, Universidad Panamericana, Augusto Rodin 498, Ciudad de Mexico 03920, Mexico

**Keywords:** agave bagasse, tequila industry, agro-industrial waste, cellulosic fibers characterization, life-cycle analysis

## Abstract

In the present research work, the use of agro-industrial waste such as agave bagasse from the tequila industry was carried out. The agave bagasse was treated to obtain biosorbent and hydrochar materials. Direct Blue 86 was used as an adsorbate model to evaluate the performance of both materials. The adsorption studies showed an adsorption capacity of 6.49 mg g^−1^ in static and 17.7 mg g^−1^ in dynamic, associated with a physisorption process between functional groups of the material and the dye. The characterization of the biosorbent showed that the material was mainly composed of macroporous fibers with a surface area <5.0 m^2^ g^−1^. Elemental analysis showed a majority composition of C (57.19 wt%) and O (37.49 wt%). FTIR and XPS analyses showed that the material had C-O, C=O, -OH, O-C=O, and -NH_2_ surface groups. RAMAN and TGA were used to evaluate the composition, being cellulose (40.94%), lignin (20.15%), and hemicellulose (3.35%). Finally, the life-cycle assessment at a laboratory scale showed that the proposed biosorbent presents a 17% reduction in several environmental aspects compared to hydrochar, showing promise as an eco-friendly and highly efficient method for the remediation of water contaminated with dye, as well as being a promising alternative for the responsible management of solid waste generated by the tequila industry.

## 1. Introduction

In recent years, the tequila industry has had an increase in the export and commercialization of “Tequila”, a regional beverage obtained by the distillation of musts. Tequila is prepared directly from material extracted from *Agave tequilana* Weber blue variety hearts, harvested in the geographic region of the same name [1]. Within the agave–tequila production chain, the main solid waste generated is agave fiber or bagasse, produced during the extraction of juice from cooked agave pineapples. It is estimated that 40% of the total weight of the agave corresponds to residual bagasse, and for every liter of Tequila produced, 1.4 kg of wet bagasse are generated [2]. That is, a single factory with a production of 30,000 L of Tequila/day can produce up to 30 tons of bagasse/day. According to data provided by the Tequila Regulatory Council (CRT, by its acronym in Spanish) in 2021, 1,866,000 tons of agave were consumed to produce Tequila [3]; considering that 40% of the weight of agave is converted into residual bagasse, it is estimated that, in parallel, 746,400 tons of agave were generated in 2021. Based on this, and considering that these residues apparently have no use, the environmental problem, and the need to produce value from this residual biomass becomes evident. Now, several processes have been proposed to evaluate the possible uses of this residue. Traditionally, the most common management and disposal practices are to use it as fuel, as a filling material in the manufacture of mattresses and seats for motor vehicles, in the manufacture of bricks, in the production of pots, as agglomerate in the manufacture of sheets for construction or furniture, and, more commonly, to simply deposit it or burn it on land near the company, which leads to contamination of the soil and the atmosphere [4]. However, it is necessary to obtain differentiated and specific products that give the material added value. In this sense, some of the recovery strategies have focused on obtaining useful products for the food industry [5,6]. Other recovery strategies have focused on the production of fuels and fertilizers [7,8,9]. Finally, one of the proposals to valorize agave bagasse that has begun to gain interest is the manufacture of advanced materials; such is the case for the proposals by Nieto-Delgado et al. [10,11,12] and Chávez-Guerrero & Hinojosa [13], who evaluated the production of activated carbon from tequila and mezcal agave bagasse by chemical activation, finding that it is feasible to obtain activated carbons that are competitive with those currently marketed for the remediation of contaminated effluents.

Although, to date, studies have made significant progress to achieve the use of waste as an adsorbent material, there is still a need to show that its synthesis and use processes represent an efficient and environmentally friendly alternative. In this sense, an alternative that has recently gained wide acceptance to evaluate how eco-friendly materials and their synthesis process are is the Life-cycle Assessment (LCA) methodology. The environmental management tool LCA follows the ISO 14040 [14] and 14044 [15] guidelines. Through this methodology, the environmental loads associated with a material can be evaluated through the quantification, modelling, and interpretation of the energy and materials used, and the waste released into the environment to determine the comparative performance of the materials and the process. Recent research works have demonstrated its potential application to assess the environmental impacts of different adsorbent materials, among which are alginate-based nanocomposite beads for the removal of Pb (II) [16], alginate waste for cadmium biosorption [17], nanoadsorbents for water remediation [18], adsorbents for fluoride removal from drinking water [19], and biosorbents from sugarcane bagasse for the of removal heavy metals [20]. All of them have achieved, through different approaches to the problem, results that demonstrate the potential of the tool.

On this basis, the present research presents the synthesis, characterization, and use of two types of adsorbents prepared from agave bagasse for their use as biosorbents in the removal of Direct Blue 86 in aqueous solution (adsorbate model). For this purpose, in the first stage, a biosorbent was prepared through a simple method consisting of a mechanical treatment that does not involve the use of specialized machinery or high energy consumption. The second material was prepared using a hydrothermal synthesis, considered to be an eco-friendly process to obtain a hydrochar. In the second stage, the characterization and use of the materials as a water filter were studied to determine the basic and design aspects in the adsorption process. Finally, in a third stage, a LCA analysis was carried out to show that the proposed process and materials are respectful of and friendly to the environment.

## 2. Materials and Methods

### 2.1. Synthesis and Characterization of Adsorbent Materials

Description of the raw material (agave bagasse): the agave bagasse used as a precursor for the adsorbent materials ws obtained from a tequila manufacturer located in the Valles region, Jalisco Mexico, which uses a diffuser extraction of sugars. This practice consists of raw ripping *Agave tequilana* Weber blue variety plants. The ripper product is transported to the diffuser, where water is added at the opposite end and filtered through the agave bed. The water dissolves the sugar in the agave, giving rise to a sugar-rich juice which is collected in a vat. This juice is pumped to the next stage of tequila production and the process is repeated until the juice reaches maximum concentration at the feed end of the diffuser. The torn and exhausted agave (poor in sugars) is removed from the process, and is called “bagasse,” which is considered a processing residue.

Biosorbent and hydrochar were prepared from agave bagasse The biosorbent material was prepared by sun-drying the agave bagasse for 24 h. Subsequently, a wash with ethanol/water solution (50/50) was carried out to remove impurities adhered to the material (24 h), followed by a second sun-drying (24 h).

Hydrochar preparation was performed as follow: In the first stage, the bagasse was sun-dryied for 24 h and washed using an ethanol/water solution (50/50) to remove impurities. Afterward, the material was dried under controlled conditions at 105 °C for 24 h in an electric oven. A shredded manual stage was carried out to separate the finest fiber from the rest of the material. The resulting biomass was subjected to a hydrothermal synthesis process that consists of placing approximately 5 g of biomass and 40 mL of distilled water inside a hydrothermal reactor. The process was carried out at a temperature of 200 °C for 72 h. The temperature ramp used was 10 °C min^−1^. Subsequently, the material was subjected to a wash with distilled water to eliminate the synthesis leachate and was dried at 105 °C for 24 h.

Finally, for both materials, a manual shred was carried out with an aluminum bristle brush to obtain homogeneous fibers with a particle size of 0.65 mm.

The morphology of the fibers was obtained by taking microphotographs of the material’s surface using a field-emission environmental scanning electron microscope (ESEM), model FEI Quanta 400 (Diepoldsau, Switzerland). At the same time, a JSM6510LVEDS microscope (Krefeld, Germany) was used to study the material’s elemental composition.

Determination of textural properties (specific surface area S_BET_ and total volume of pores) were carried out by obtaining nitrogen adsorption isotherms using a QUADRASORB-SI equipment from the Quantachrome Ins. at −196 °C.

The surface chemistry of the material was studied by infrared spectroscopy (FTIR), RAMAN spectroscopy, and X-ray photoemission spectroscopy (XPS). FTIR studies were performed in the region of 400 to 4000 cm^−1^ using a Bruker Tensor 27 spectrophotometer equipped with an ATR module. RAMAN spectroscopy studies were performed using a JASCO NRS-5100 Dispersive micro-Raman microspectrophotometer with a red laser wavelength of 780 nm, and a power of 4 mW in a range of 100 to 3000 cm^−1^. Finally, the X-ray photoemission spectroscopy (XPS) studies were carried out using an ESCA 5701 machine from Physical Electronics. XP spectra were obtained with a monochromatic Al Kα X-ray source (1486.71 eV) with operational conditions of 150 W, 15 kV, and 10 mA at a pressure of 3 × 10^−8^ Torr in the chamber. The scan spectra were determined in an energy range of 0–1100 eV with a step energy and size of 80 eV and 1 eV. Once the peaks were examined, high-resolution scans with a step energy of 40 eV and a step size of 0.05 eV were performed. Each spectral region of interest was scanned several times to obtain good signal-to-noise ratios. To obtain the number of components, the position of each peak, peak areas, and the resultant spectra after background signal correction were fitted to the Lorentz and Gauss curves (Voigt profile), in which assignments of peaks were done according to the existing literature.

Finally, the physical composition of the material was studied by thermogravimetric analysis using a METTLER-TOLEDO model TGA/DSC1 thermogravimetric analyzer. The experiments were carried out in an air atmosphere with a flow of 100 mL min^−1^. The initial mass of the material was approximately 40 mg for all experiments. The temperature range was from 28 to 900 °C and the heating rate was 0.2 °C min^−1^. From these conditions, the weight loss of the material was recorded as a function of temperature.

### 2.2. Adsorption Studies

Direct Blue 86 dye (DB-86) is a phthalocyanine-type and azo dye which is widely used in the textile industry. The physicochemical properties are widely described in Table S1.

Adsorption studies were performed using a batch adsorber (glass vials) in which 50 mL of DB-86 solution was placed. A mass of 0.40 ± 0.01 g of adsorbent was added to each vial. The vials were sealed and stirred magnetically at 200 rpm. To determine the effect of pH on the adsorption process, studies were carried out at pH 2, 4, 6, and 8 (Supplementary Figure S1). Having chosen the best condition (pH = 2), studies were carried out to evaluate kinetics (t vs. q_t_) and adsorption equilibrium (C_e_ vs. q_e_) at different temperatures: T_1_ = 25 °C, T_2_ = 35 °C, and T_3_ = 45 °C.

The concentration of DB-86 present in the aqueous solution was determined using a Shimadzu UV-Vis spectrophotometer at a wavelength of 628 nm. Adsorption capacities of the adsorbents (q, mg g^−1^) were determined from a mass balance (Table S2).

The kinetic adsorption models used to study the adsorption rate of the experimental data were Elovich, pseudo-first order, and pseudo-second order (Table S2) [21]. For these experiments, an initial concentration of 50 mg L^−1^ was used.

The adsorption data obtained were correlated to the equilibrium isotherm models: Langmuir, Freundlich, and Temkin (Table S2) [21,22]. For these experiments, DB-86 solutions were used in a concentration range from 50 to 550 mg L^−1^.

**Figure 1:**
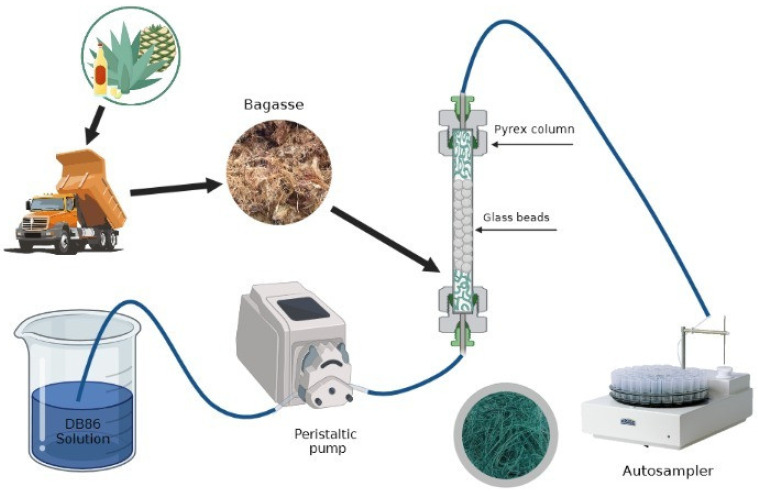
Experimental setup for fixed-bed column study.

After the evaluation of the adsorbent materials in static studies, the continuous flow study was carried out using a fixed-bed column. The fixed-bed column system was conducted in a laboratory-scale glass column using a peristaltic pump, a 2 L recipient for the feeding solutions, and an autosampler at the column outlet. The specifications of the glass columns were a height of 14 cm and an internal diameter of 12 mm, respectively, packed with an amount of biosorbent (0.4 g = 1 cm of height); the rest was filled with inert glass spheres. Feeding solutions with initial dye concentrations of 5–50 mg L^−1^ were pumped into the column by a peristaltic pump with a flow rate at 2.5 mL min^−1^ (Figure 1). Effluent samples were collected at defined time intervals and their concentration was determined by Ultraviolet-Visible spectroscopy (UV-1800, Shimadzu, Kyoto, Japan).

From the breakthrough curves obtained, breakthrough time (t_b_), saturation time (t_s_), height of the mass transfer zone (H_MTZ_), adsorption capacity of the column at the breakthrough point (q_b_), and the saturation time (q_s_) were measured [23]. Additionally, the kinetic models of Adams-Bohart and Thomas were studied to analyze the breakthrough curves of dye adsorption in the continuous mode. The equations used for each determination are presented in Supplementary Table S2.

Adsorption models (kinetic and isotherm) were analyzed by using non-linear equations, using the Levenberg-Marquardt algorithm as an estimation method (convergence criterion: 1.0 × 10^−6^).

### 2.3. Life Cycle Assessment (LCA) Study

The LCA study followed the guidelines of the ISO 14040 [14] and 14044 [15] standards. The methodological framework followed is described below.

First phase: definition of the objective and scope of the particular LCA for the assessment of the environmental performance of adsorbents. The objective of this LCA study was to comparatively evaluate the potential environmental impacts of two adsorbents synthesized in the laboratory through different processes, that is, in two different ways. The scope of the study included the synthesis stages of the adsorbents and their respective use on a laboratory scale. The selected functional unit was of the physical type and was 1 kg of agave bagasse for the synthesis processes and the use of the respective adsorbent obtained. That is, the comparison was made from the same biomass.

For the Life Cycle Impact Assessment (LCIA), the ReCiPe method was selected for easy interpretation and scientific robustness. This method can significantly reflect the potential environmental impact of the system under analysis through its varied and characteristic impact categories. RIVM, CML, PRé Consultants, Radboud Universiteit, Nijmegen, and CE Delft created the ReCiPe methodology [24]. ReCiPe was developed to combine the advantages of the CML2001 (scientific soundness) and Eco-Indicator99 (ease of interpretation) methods. It is internationally accepted and is considered the successor of the previous methodologies. It integrates the environmental problem-oriented and the damage-oriented approaches. The categories selected in this research, according to the type of expected impacts, were climate change, fossil depletion, freshwater consumption, human toxicity, land use, photochemical ozone formation, and terrestrial ecotoxicity.

At last, the GaBi software was used to model the potential environmental impacts. It is one of the world’s leading LCA softwares. GaBi was developed by PE INTERNATIONAL in Germany and is now supported by Sphera.

Second phase: life cycle inventory; third phase: life cycle impact assessment. The information on the reference flows of both the process and the use consists of real data and comes from the laboratory. In addition, the ecoinvent Life Cycle Inventory (LCI) database included in Gabi was used as a complement to the information on the loads associated with the process. The geographical context of Mexico influences a predominantly fossil energy matrix. The LCI was modeled using the ReCiPe midpoint method in Gabi Software. The impact categories considered were mentioned above.

All currents associated with energy and water were considered, except for the water to be purified in the stage of use. The water to be purified and the water already purified were excluded. In the stage of use, a greater amount of purified water was synonymous with a good physicochemical performance of the adsorbent (since it takes longer to saturate and purifies more water); however, from the point of view of the LCA, a greater amount of material usually indicates worse environmental performance. The Direct Blue 86 (adsorbate model) was considered in terms of the adsorbent saturation and the final disposal.

**Figure 2:**
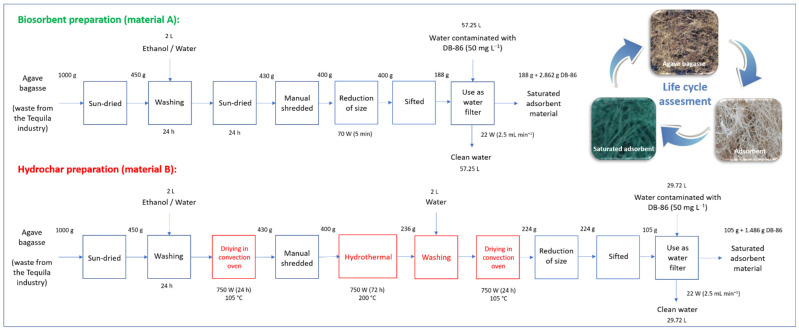
Life cycle diagram of production of adsorbent materials from agave bagasse.

The block diagram of Figure 2 shows both the stages of the synthesis process and the use of the two adsorbents that were studied. In addition, the real data of the reference flows used for the elaboration of the LCI are analyzed and discussed. The electrical equipment data (powers and consumptions), emissions, waste, and discharges are not included in the graph.

## 3. Results and Discussion

### 3.1. Characterization of the Adsorbent Materials

**Figure 3:**
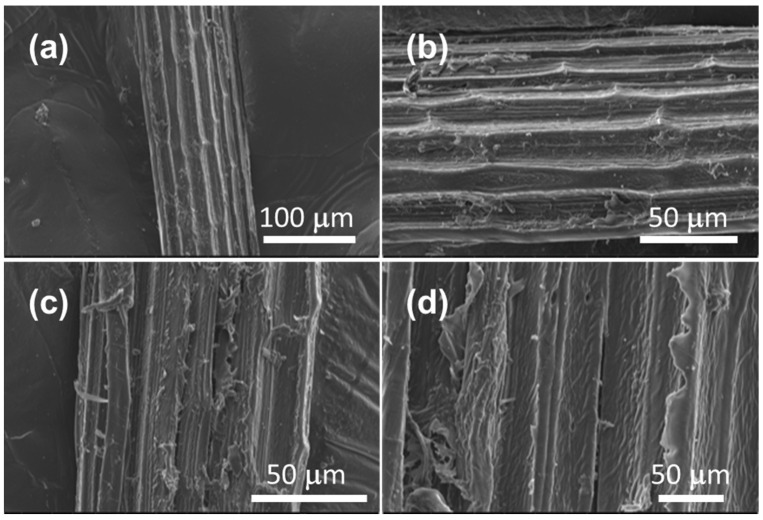
Microphotographs of the biosorbent prepared from agave bagasse at different magnifications: (**a**) 100 μm (**b**) 50 μm (**c**) 50 μm (**d**) 50 μm.

The most representative microphotographs of the biosorbent obtained are shown in Figure 3. The images show an assembled structure in several layers; similar results have been previously reported by other authors [25,26,27]. Well-defined channels interconnected along the fiber were also observed. The length of the fibers was 10 cm on average, while the diameter was 108.00 ± 6.55 μm. Through EDX spectroscopy analysis, it was possible to obtain a semi-quantitative reading of the elemental composition of the material, showing a content of mainly carbon (57.19 wt.%) and oxygen (37.49 wt.%), associated with the high amount of cellulose in the material. The rest of the composition included traces of Calcium (2.03 wt.%), Nitrogen (2.09 wt.%), Sodium (0.53 wt.%), Potassium (0.14 wt.%), Magnesium (0.22 wt.%), Aluminum (0.17 wt.%), and Silicon (0.14 wt.%), which were attributable to the minerals present in the soil where the agave was grown. It is important to highlight that the hydrogen composition is not presented because EDX analysis does not allow its quantification.

The nitrogen adsorption isotherm at −196 °C was obtained to determine the textural properties of agave fiber (Figure S2). It was concluded that the materials are essentially meso- and macro-porous, since the surface area (S_BET_) was <5.00 m^2^ g^−1^; this value is within the range previously reported for similar materials of the same origin [28]. A low value of the surface area was due to the null existence of microporosity in the material, in such a way that the total volume of pores (V_0_._95_ = 0.002 cm^3^ g^−1^) can be expressed as the volume due to the presence of meso- and micro-porosity.

For the case of the hydrochar, the results are presented in detail in the supplementary material. The SEM micrographs are shown in Supplementary Figure S3. It was observed that the fibers mostly preserved the morphological characteristics of the precursor material, the most evident alteration corresponding to the exfoliation of the material (Figure S3c), which can be associated with the extreme conditions of temperature and pressure that they were exposed to. This modification caused the material to have a total pore volume of V_0_._95_ = 0.004 cm^3^ g^−1^. Finally, EDX studies showed that the hydrochar contained carbon (72.1 wt.%), oxygen (26.9 wt.%), potassium (0.2 wt.%), calcium (0.2 wt.%), and magnesium (0.1 wt.%).

**Figure 4:**
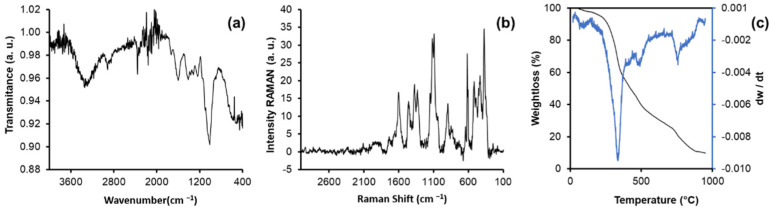
(**a**) FTIR spectrum (black line), (**b**) RAMAN spectrum (black line), and (**c**) Thermogravimetric analysis of biosorbent prepared from agave bagasse. DTG (blue line) and TGA (black line). Figure 4b shows the RAMAN spectrum of the agave fiber after baseline correction and area normalization. Each of the bands was studied in detail and compared with information previously reported in the literature. The bands at 2945, 1658, 1620, 1601, 1423, 1330, 1270, 1130, 786, 730, 634, 595, and 560 cm^−1^ can be attributed to the different stretching, bending, deformation, and torsion vibrations of the bonds related to lignin, whilehile the bands centered at 2897, 1480, 1465, 1378, 1121, 1098, 1037, 896, 520, 458, 435, and 378 cm^−1^, can be attributed to the different vibrations of the cellulose and hemicellulose structure [7].

The FTIR spectrum of the fibers is presented in Figure 4a. An absorption band can be seen in the region of 3342 cm^−1^, which indicates the presence of -OH bonds, and the band centered at 2916 cm^−1^ can be attributed to the presence of -CH and -CH_2_ bonds, while the band at 1600 cm^−1^ can be attributed to COOH bonds. Finally, the bands at 1238 cm^−1^ and 1012 cm^−1^ can be associated with C-O-C and C-O bonds, respectively. All the functional groups described above can be related to the structure of cellulose, pectin, and lignin [29].

Figure 4c shows the thermogravimetric analysis, in which the thermogram for the obtained biosorbent is shown in black. To visualize the stages of thermal degradation with better clarity, the derivative of the function with respect to temperature (dw/dT) was obtained. Through this mathematical analysis, it is possible to appreciate the five stages (peaks) during the combustion of the material in a similar way to other agro-industrial by-products [30]. The first stage occurs between 20 and 140 °C and represents the loss of water and moisture present in the material. The second stage is between 140 and 227 °C and can be attributed to the degradation of hemicellulose. At this stage, the fiber loses approximately 5.70% of its initial weight. The third stage of thermal decomposition occurs between 227 and 400 °C, this stage is where the greatest weight loss occurs (approximately 46.64%) due to the oxidation of the cellulose present. A fourth stage occurs between 400 and 580 °C where lignin degradation occurs (66.79%). In the fifth stage, between 580 and 790 °C, the degradation of CaCO_3_ to CaO and CO_2_ occurs, both of which degrade at a relatively slow rate [31]. Finally, after this temperature, it is observed that the weight begins to be constant and acquires a value of approximately 4 mg (10% of the initial mass). The sample obtained at this temperature is white, indicating the presence of Calcium and traces of more inorganic elements such as Sodium, Potassium, Magnesium, Aluminum, and Silicon. Based on the above data, Table 1 was constructed, which summarizes the composition of the sample.

On the other hand, the physicochemical characterization of hydrochar prepared from agave bagasse is presented in Supplementary Figure S4. FTIR and RAMAN spectroscopy studies did not show significant differences with respect to the prepared biosorbent. It was observed that, in the material, the functionalities were mostly -OH, -CH, -CH_2_, -COOH, C-O-C, and C-O groups. However, the DTG studies of the hydrochar show significant changes when compared to the biosorbent material. Four fundamental stages were observed during the thermal degradation of the material. The first one between 30 and 150 °C, where dehydration reactions occur. The second stage, in a range from 200 to 400 °C, is associated with the formation of char. In this stage, dehydration and decarboxylation reactions take place, eliminating oxygen that is present in the organic matter in the form of water and CO_2_ [32,33]. Due to the above, an increase in the amount of carbon can be seen compared to the biosorbent. The third stage, which occurred between 400 and 550 °C, is attributed to the oxidation of the solid carbonaceous material. Finally, the fourth stage, between 550 and 600 °C, is associated with the complete degradation of the material, leading to the formation of ashes, corresponding to 5.9% by weight.

**Figure 5:**
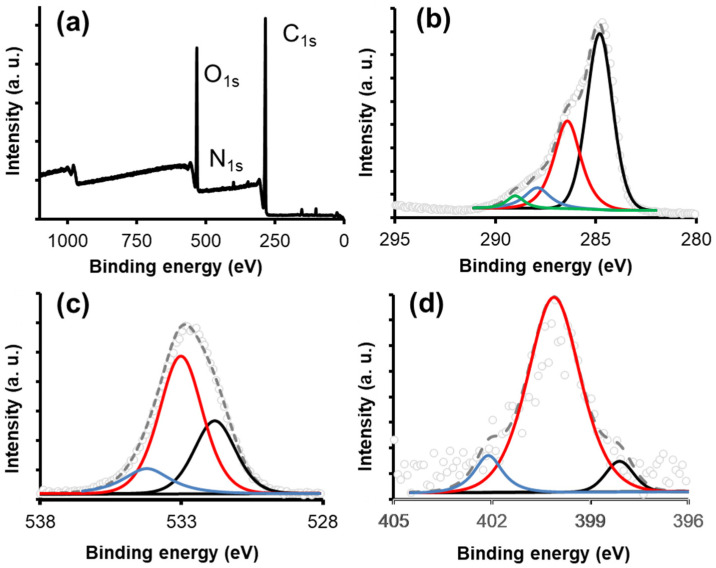
Deconvolution of the XP spectra of the agave bagasse. (**a**) XPS survey spectra, (**b**) C_1s_ XPS spectrum: C-C bond (black line), C-O bond (red line), C=O bond (blue line), and O-C=O bond (green line) (**c**) O_1s_ XPS spectrum; C=O bond (Black line), C-O bond (red line), and O-H bond (blue line), and (**d**) N_1s_ XPS spectrum: Amide bond (black line and red line) and NH^3+^ bond (blue line). Experimental data (dotted line), fitting curve (Dashed line).

X-ray photoemission spectroscopy (XPS) was used to characterize the surface chemistry of the sample on its outermost surface. This analysis, together with the FTIR, EDX, and RAMAN techniques, allowed for a complete panorama of the surface chemistry, the chemical composition of the material, and the distribution of the different surface groups. The wide scan spectrum (0–1100 eV) showed that the signals associated with the C_1s_, O_1s_, and N_1s_ regions were predominant (Figure 5a), with small traces of the elements described in the previous sections. Due to this, these regions were analyzed in high resolution. For the quantitative analysis (Table 2), the sensitivity factors recommended by the manufacturer were used: 0.205 for C_1s_, 0.66 for O_1s_, and 0.42 for N_1s_. The high-resolution spectrum of the C_1s_ region (Figure 5b) was decomposed into four different species, similar to what has been previously reported for materials of the same origin [34,35,36].

The first peak, centered at 284.79 eV, is associated with C-C bonds. The second peak (286.40 eV) corresponds to C-O and/or C-O-C bonds. The third peak (287.90 eV) can be attributed to the presence of C=O bonds. Finally, the fourth peak (289.00 eV) can be related to O-C=O structures. The oxygenated groups described above were corroborated by performing the decomposition of the high-resolution spectra of the O_1s_ region (Figure 5c). Three peaks centered at 531.80, 533.0, and 534.20 eV were observed, corresponding to the bonds of the C=O, C-O, and –OH groups, respectively. Finally, the high-resolution spectrum of the N_1s_ region is shown in Figure 5d; this was decomposed into three peaks centered at 398.10, 400.10, and 402.10 eV, attributable to amide and NH^3+^ groups.

Finally, as part of the life cycle assessment studies, the yields in the preparation of both materials were determined. Starting from 1 kg of bagasse from the tequila industry, it is possible to obtain 188 g of biosorbent and 105 g of hydrochar, that is, a yield of 18.8% and 10.5%, respectively. These calculations and those corresponding to performance in fixed-bed water treatment are presented in detail in Figure 2 and correspond to the fundamental information used for the life cycle assessment study of adsorbents.

### 3.2. Adsorption Studies of DB-86 onto Carbon Materials

**Figure 6:**
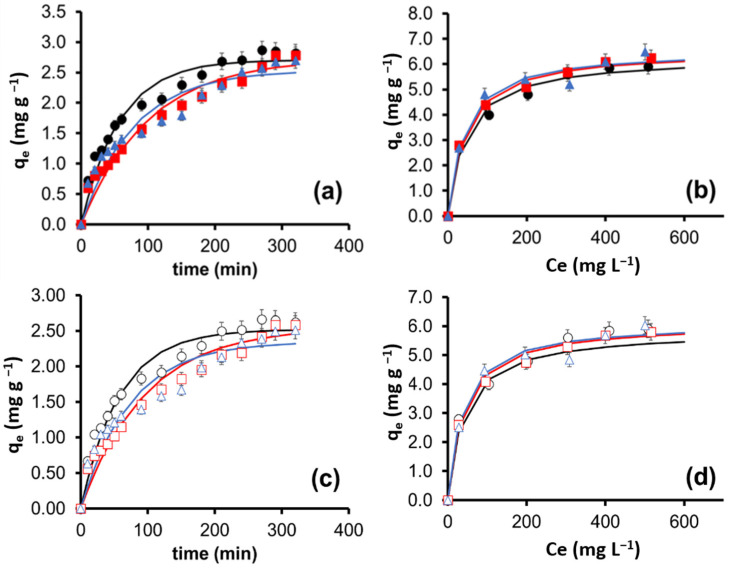
Batch adsorption studies of DB-86 onto adsorbents prepared from agave bagasse fibers: biosorbent material: (**a**) Adsorption kinetics studies, (**b**) Adsorption equilibrium studies; hydrochar material: (**c**) Adsorption kinetics studies, (**d**) Adsorption equilibrium studies. Experimental conditions: 0.40 ± 0.01 g of adsorbent, 50 mL of adsorbate, pH 2, 200 rpm, equilibrium time 180 min, temperature for biosorbent material: ● 25 °C, ■ 35 °C, and ▲ 45 °C. Temperature for hydrochart ○25 °C, □ 35 °C, and △ 45 °C.

Adsorption kinetics studies are shown in Figure 6a,c. At the beginning of the process for both materials, an exponential increase in the adsorption capacity was observed, reaching 62% of the total adsorption capacity at 60 min. This effect can be attributed to the fact that adsorption is carried out first in the most exposed active sites of the carbon materials, then, due to diffusional phenomena, the adsorbate has access to the less accessible sites, achieving the adsorption equilibrium [37]. This can be seen in the asymptotic form of the graph in which 90% adsorption capacity is reached at 180 min. The adsorption mechanism can be correctly described by the pseudo first order model (Table 3), being the one that best predicts the adsorption capacity (q_calc_). Due to the above, it is concluded that the predominant adsorption mechanism is the physisorption between the adsorbate–adsorbent with a rate constant 0.013 ± 0.003 min^−1^ [38,39].

The experimental adsorption equilibrium data are shown in Figure 6b,d. From these data, it is possible to determine the maximum adsorption capacity of the materials: 6.57 mg g^−1^ and 6.12 mg g^−1^ for biosorbent and hydrochar, respectively. Likewise, it was concluded that the Langmuir model is the one that best describes the adsorption process attributable to the formation of a monolayer of adsorbate on the surface of the adsorbent (Table 4) [40].

The study of the effect of pH on the adsorption capacity is presented in Supplementary Figure S1. The effect of pH (2, 4, 6, 8, and 10) was evaluated at different temperatures (25, 35, and 45 °C). It was concluded that the best condition for the adsorption process is a pH of 2. It is proposed that this phenomenon is related to the fact that the DB-86 dye in acid solution behaves as an anionic dye. In such a way, the R-SO^3-^ bonds remain with a partial negative charge, which is attracted by the materials surfaces that are protonated due to the absorption of H^+^ from the medium, as discussed later.

A comparison of the results obtained against those previously reported for the removal of DB-86 present in water are presented in Table 5, in which it is highlighted that the biosorbent material obtained in the present study from agave fibers, since its preparation is simple, is respectful to the environment (as will be discussed later) and provides competitive adsorption capacities against activated carbons.

On the other hand, isotherm curves obtained at different temperatures of DB-86 adsorption onto agave fibers were obtained at the temperatures of 25 °C (298.15 K), 35 °C (308.15 K), and 45 °C (318.15 K) at pH 2. For the determination of the thermodynamic equilibrium constant of adsorption, the Langmuir equilibrium constant, *K*, was used to evaluate the adsorption enthalpy change (∆*H_ads_*) [37,46,47] in Equation (1):

(1) $$K=K_{0}e^{\Delta H_{ads}/RT}$$

The experimental values of *K* were fitted in this equation, and the enthalpy of adsorption was estimated to be −5.095 kJ mol^−1^ (R = 0.982) and −4.689 kJ mol^−1^ (R = 0.981) for biosorbent and hydrochar, respectively. The negative value of Δ*H_ads_* implies an exothermic behavior. 

From the above results, it can be concluded that the adsorption mechanism present in the process is associated with physisorption phenomena between the adsorbent and adsorbate with a binding energy of −5.60 kJ mol^−1^. It is proposed that this phenomenon is related to the fact that the DB-86 dye in acid solution behaves as an anionic dye due to the solubility of the Na^+^ cation in water. In such a way, the R-SO_3_^−^ bonds remain with a partial negative charge which is attracted by the C-O, C=O, O-C=O, and -OH groups that are protonated (positive partial charge) due to the absorption of H^+^ from the medium [42,43,48]. Due to the above, at pH > 2, the capacity adsorption capacity decreases considerably, since the previous mechanism is not favored (Supplementary Figure S2). On the other hand, the adsorption studies showed, in all cases, that the hydrochar shows a decrease in adsorption capacity of 7% with respect to the biosorbent, which is attributable to the decrease in oxygenated sites in the material (Figure 2). In this way, it is confirmed that the adsorption mechanism of DB-86 in the proposed materials is due to physisorption phenomena between the negatively charged material and the cationic dye.

**Figure 7:**
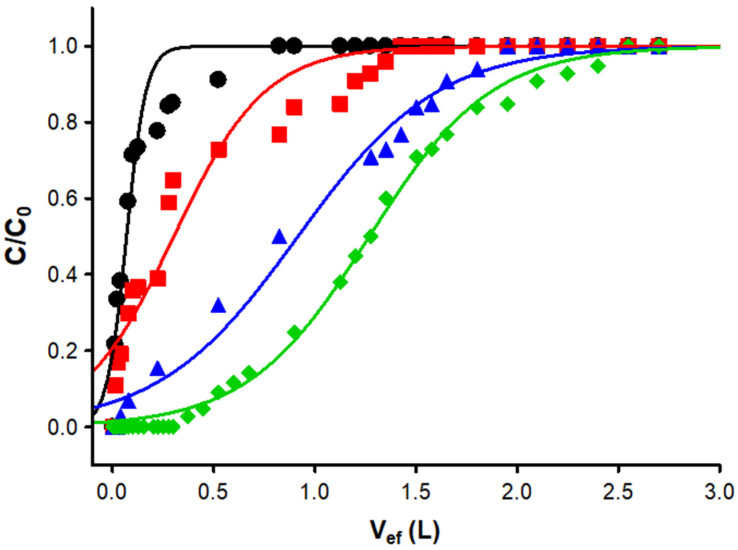
Breakthrough curves obtained from adsorption tests in a continuous flow using 0.4 g of adsorbent (Z = 1 cm), 2.5 mL min^−1^ y C_0_ = ● 50 mg g^−1^, ■ 25 mg g^−1^, ▲10 mg g^−1^ y, and ♦ 5 mg g^−1^.

Finally, continuous flow adsorption studies were carried out using a fixed-bed column to show the prepared material’s potential in water treatment processes. The breakthrough curves obtained at different initial concentrations are shown in Figure 7. To consider that the column was saturated, a criterion of C/C_0_ > 0.9 was considered, and, for the breakthrough time, the condition was C/C_0_ > 0.1. Once the rupture curves were constructed, the adsorption capacity of the fixed bed (q_total_), the rupture time (T_b_), and the height of the mass transfer zone (H_MTZ_) [49,50] were determined for the experimental conditions which were tested (Table 5).

It was observed that the maximum adsorption capacity at the conditions tested was 17.7 mg g^−1^. When comparing these results against those obtained in the batch studies, it was concluded that the adsorption capacity increased significantly. Similar behaviors have been previously reported for the adsorption of dyes such as methylene blue [51], acid blue 29 [52], and crystal violet [53]. This effect can be attributed to the pretreatment that the adsorbent receives when packed inside the column. Since it is important to highlight this as a fundamental step in continuous adsorption studies, it is necessary to pass a flow of water (without the presence of the adsorbate) to the same temperature in which the column will be operating in order to correctly pack the material and detect possible leaks in the system. In the present study, these hydraulic tests were carried out using distilled water at 45 °C using a flow of 2.5 mL min^−1^ and a total volume of 1 L. The above process allowed the authors to obtain the surface of the adsorbent hydrophilic properties, since the moisture in the material improves the contact area between adsorbent—adsorbate, achieving improvements in the adsorption capacity of the dye, matching what has been widely reported in the dyeing process of natural cellulose fibers [54].

Finally, the experimental column data were fitted well with the Thomas kinetic model (R2 > 0.99), predicting a better fixed-bed column performance than the Adams–Bohart model (Table 6). Therefore, the previously exposed mechanisms are corroborated, since the Thomas model assumes that adsorption is not controlled by a chemical reaction between adsorbent—adsorbate but by mass transfer at the interface [50].

### 3.3. Life Cycle Assessment of the Adsorbent Materials

**Figure 8:**
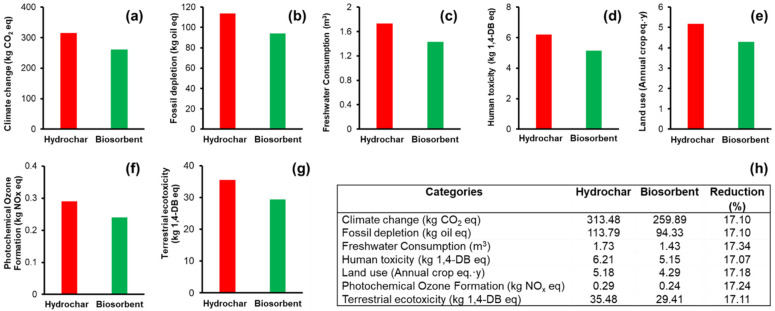
Life-cycle assessment of production of adsorbent materials from agave bagasse. Categories evaluated: (**a**) climate change, (**b**) fossil depletion, (**c**) freshwater consumption, (**d**) human toxicity, (**e**) land use, (**f**) photochemical ozone formation, (**g**) terrestrial ecotoxicity, and (**h**) comparative table between the environmental impacts of hydrochar vs. biosorbent.

To estimate and evaluate the impacts that the proposed biosorbent may have on the environment during the stages of synthesis and use, the LCA was carried out. In the same way, the LCA of hydrochar was developed. The differences in the synthesis processes and yields in the adsorption capacity of DB-86 were presented previously in Figure 8. The LCA results of both adsorbents were contrasted to obtain the real environmental impacts and assert which adsorbent has a better environmental performance. The obvious expectation was proven. The synthesis process with fewer stages is the process that has the lesser environmental impact, being the biosorbent process.

The diagrams created in the GaBi software for the life cycle impact assessment of adsorbents can be seen in Supplementary Figure S5. In them, the reference flows and currents considered are shown. On the other hand, Figure 8 shows the LCA results presented in seven bar graphs representing the seven impact categories modeled. The respective comparisons between the environmental performance of the studied adsorbents can be observed. In all of them, a clear trend of a greater impact on hydrochar is shown, as well as a consequent lower impact of biosorbent. Additionally, an impact results table is displayed. It shows the value of the impact categories and their units.

The environmental impact reduction in all categories is around 17%. Usually, there are no trends in the performance of the different categories of environmental impact in the typical LCA study results. However, on this occasion, trends were presented. This can possible be attributed to the relatively few-step synthesis (in terms of a few processes), and to limited currents or material flow (in terms of a few materials). However, this does not mean that the environmental impact loses representativeness or can be minimized. What is relevant is that there is a lower impact of the biosorbent.

The decrease in the impact that the biosorbent presents in reference to hydrochar is attributed to the elimination of some of the processes which use electrical consumption such as oven drying and hydrothermal. Drying in the sun is essential to reducing the impact of this material. The Mexican energy matrix, where fossil energy prevails, must be considered responsible for the high impact of the hydrochar, obviously considering that this adsorbent has more stages in its process. Therefore, the method of preparing the biosorbent is more relevant. The biosorbent synthesis is consistent with the actual trend of decarbonization of processes.

It is important to highlight the climate change impact category; therefore, the following relationship is presented. The biosorbent prepared to start from 1 kg of agave bagasse, purifies 57.25 L (as mentioned above) and performs environmentally with a reduction of 54 kg of CO_2_ eq compared to hydrochar, which also only purifies 29.72 L before its saturation. The 54 kg of CO_2_ is a relevant result. To give context to that number, the EPA Greenhouse Gas Equivalence Calculator [55] was used. The 54 kg of CO_2_ eq that the biosorbent does not emit in its preparation and use process are comparable to the emission of an average car travelling 215 km.

## 4. Conclusions

It is possible to obtain a natural adsorbent material (biosorbent) from agave bagasse (solid residue from the tequila industry) using a simple process with better adsorption performance compared to hydrochar. Adsorption studies show its potential application as a water filter. From an eco-friendly point of view, biosorbent also performs better than hydrochar. Although, both come from waste management processes from the tequila industry, the differences in their synthesis process and their yields influence decision-making focused on minimizing the environmental impact. The biosorbent process has a higher yield and its use is much more efficient than its hydrochar counterpart.

From the life cycle analysis studies, it was concluded that the impacts associated with fossil fuels and their effect on both climate change and photochemical ozone prevail. The above is a consequence of a predominantly fossil energy matrix. From the point of view of the decarbonization of biosorbent processes, the studied process presents a significant improvement of 17% compared to hydrochar. Based on the previous studies, the use of solid waste from the tequila industry is proposed as a raw material for the preparation of natural adsorbent materials to promote actions that will lead the industry to migrate to a circular economy.

## Figures and Tables

**Table 1:** Agave fiber composition obtained from the thermogravimetric analysis.

Temperature Range (°C)	Composition	(%)
20–140	humidity and water	2.35
140–227	hemicellulose	3.35
227–400	cellulose	40.94
400–580	lignin	20.15
580–790	CaCO_3_ decomposition	15.73
790–950	inorganic compounds	17.47

**Table 2:** Binding energies (eV) of the C_1s_, O_1s_, and N_1s_ regions obtained from the mathematical analyses of the XP spectra.

C_1s_	Bond	FWHM (eV)	% Peak	O_1s_	Bond	% Peak	%O	N_1s_	Bond	% Peak	%N
284.79	C-C	1.52	58.07	531.80	C=O	29.26	18.28	398.10	Amide	6.84	0.54
286.40	C-O		31.59	533.00	C-O	58.27		400.10	Amide	83.98	
287.90	C=O		7.39	542.20	O-H	12.46		402.10	NH^3+^	9.17	
289.00	O-C=O		2.93								

**Table 3:** Kinetic constants of the mathematical models proposed to describe the adsorption process of DB-86 on the adsorbent prepared from agave bagasse fibers.

Material	T	q_e, exp_	Pseudo First Order			Pseudo Second Order			Elovich		
			k_1_	q_e, calc_	R	k_2_	q_e, calc_	R	a	b	R
	(°C)	(mg g^−1^)	(min ^−1^)	(mg g^−1^)		(g mg^−1^ min^−1^)	(mg g^−1^)				
Agave fiber	25	2.80	0.0174	2.71	0.9778	0.0061	3.23	0.9911	3.103	0.0489	0.9971
	35	2.80	0.0099	2.73	0.9809	0.0026	3.55	0.9878	2.312	0.0193	0.9930
	45	2.70	0.0128	2.53	0.9562	0.0045	3.10	0.9733	3.000	0.0301	0.9861
Hydrochar	25	2.60	0.0173	2.52	0.9762	0.0066	3.01	0.9898	3.339	0.0455	0.9965
	35	2.60	0.0104	2.55	0.9814	0.0030	3.30	0.9885	2.513	0.0190	0.9936
	45	2.51	0.0136	2.35	0.9587	0.0050	2.88	0.9755	3.249	0.0285	0.9879

**Table 4:** Equilibrium constants of the mathematical models proposed to describe the adsorption process of DB-86 on the adsorbent prepared from agave bagasse fibers.

Material	T	q_max exp_	Langmuir Model			Freundlich Model			Temkin Model		
			q_max_	K	R	K_f_	n	R	A_T_	b_T_	R
	(°C)	(mg g^−1^)	(mg g^−1^)	(L mg g^−1^)					(L mg^−1^)	(J mol^−1^)	
Agave fibers	25	5.91	6.29	0.022	0.990	1.19	3.79	0.997	0.393	2193.50	0.992
	35	6.24	6.56	0.023	0.996	1.30	3.90	0.997	0.396	2158.64	0.998
	45	6.49	6.57	0.025	0.990	1.37	4.02	0.985	0.416	2226.42	0.963
Hydrochar	25	5.49	5.83	0.024	0.991	1.13	3.81	0.998	0.422	2358.60	0.992
	35	5.80	6.10	0.025	0.996	1.23	3.91	0.997	0.426	2321.17	0.998
	45	6.03	6.12	0.027	0.990	1.30	4.02	0.986	0.447	2394.00	0.963

**Table 5:** Comparison of adsorption capacities of DB-86 in aqueous solution on different adsorbent materials.

Material	q_(max)_ (mg g^−1^)	Reference
Biosorbent (Agave Fibers)	6.57	Present Work
Manioc husk activated carbon	6.20	[41]
Merck commercial activated carbon	3.70	
Orange peel activated carbon	33.78	[42]
Peanut shell activated carbon	21.60	[43]
Zeolitic Imidazolate Frameworks (ZIF-8)	128.00	[44]
Zeolitic Imidazolate Frameworks (ZIF-67)	230.00	
Zeolitic Imidazolate Frameworks (ZIF-8@ZIF-6)	301.69	
Granular particles of heat treated-lyophilized biomass of Neonectria radicicola	227.1	[45]

**Table 6:** Parameters of the studied models of breakthrough curves for DB-86 on column fixed bed.

C_0_	q_s_	q_b_	T_b_	H_MZT_	Adams-Bohart			Thomas		
					K_AB_	N_0_	R	K_th_	q_0_	R
mg L^−1^	mg g^−1^	mg g^−1^	min	cm	L mg^−1^ min^−1^	mg L^−1^		mL mg^−1^ min^−1^	mg g^−1^	
5	13.58	0.00	---	1.00	1.15 × 10^−5^	8.58 × 10^4^	0.6653	1.03	8.64	0.9678
10	23.18	0.69	5	0.96	3.37 × 10^−5^	4.58 × 10^4^	0.8032	0.44	18.91	0.9736
25	18.99	4.96	125	0.73	1.44 × 10^−4^	2.01 × 10^4^	0.8842	0.72	22.90	0.9943
50	15.23	7.22	240	0.75	4.39 × 10^−4^	1.06 × 10^4^	0.9144	1.66	15.85	0.9980

## Data Availability

The data that support the findings of this study are available from the corresponding author upon reasonable request.

## Supplementary Materials

The following supporting information can be downloaded at: https://www.mdpi.com/article/10.3390/ma16010008/s1, Table S1. General characteristics of Direct Blue 86. Table S2. Mathematical models used to describe the adsorption process in batch and fixed bed. Figure S1. Effect of adsorption capacity as a function of pH in solution: ● 25 °C, ■ 35 °C, ▲ 45 °C. Figure S2. Nitrogen adsorption isotherms at −196 °C for: (a) biosorbent and (b) hydrochar. Figure S3. Microphotographs of the hydrochar prepared from agave bagasse at different magnifications. Figure S4. (a) FTIR spectrum, (b) RAMAN spectrum, and (c) Thermogravimetric analysis of hydrochar prepared from agave bagasse. Figure S5. Diagrams created in the Gabi software for the life cycle impact assessment of both absorbents.
