# Supplementary material for: Evaluation of the Environmental Performance of Adsorbent Materials Prepared from Agave Bagasse for Water Remediation: Solid Waste Management Proposal of the Tequila Industry
Source: Materials (Basel). 2022 Dec 20;16(1):8. doi: 10.3390/ma16010008 (PMC9821227; doi:10.3390/ma16010008)
Supplement: Supplementary file 1 [file materials-16-00008-s001.zip › materials-2036758-supplementary.pdf]

materials

Article

Evaluation of environmental performance of adsorbent materials prepared from agave bagasse for water remediation: Solid waste management proposal of the Tequila Industry

Camila S. Gómez-Navarro [1], Walter M. Warren-Vega [1], Juan C. Serna-Carrizales [2], Ana I. Zárate-Guzmán [1, 2,*], Raúl Ocampo-Pérez [2], Francisco Carrasco-Marín [3], Virginia H. Collins-Martínez [4], Joaquina Niembro-García [5], Luis A. Romero-Cano [1,*]

[1] Grupo de Investigación en Materiales y Fenómenos de Superficie. Departamento de Biotecnológicas y Ambientales, Universidad Autónoma de Guadalajara, Av. Patria 1201, C.P. 45129 Zapopan, Jalisco, Mexico
[2] Centro de Investigación y Estudios de Posgrado, Facultad de Ciencias Químicas, Universidad Autónoma de San Luis Potosí, San Luis Potosí 78260, Mexico
[3] Grupo de Investigación en Materiales de Carbón, Facultad de Ciencias, Universidad de Granada, Av. Fuente Nueva s/n, Granada C.P. 18071, Spain
[4] Centro de Investigación en Materiales Avanzados (CIMAV), S.C. Miguel de Cervantes #120, Complejo Industrial Chihuahua, Chihuahua, Chih., 31136 Mexico
[5] Facultad de ingeniería, Universidad Panamericana, Augusto Rodin 498, Ciudad de Mexico, 03920, Mexico
* Correspondence: A.I.Z.-G. (aizg_08@hotmail.com); L.A.R.-C. (luis.cano@edu.uag.mx)

SUPPLEMENTARY MATERIAL

Table S1. General characteristics of Direct Blue 86.

**Chemical structure**	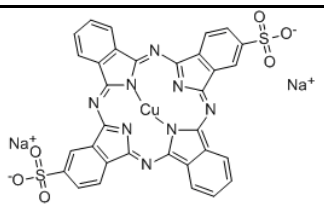
**Colour Index Number**	74180
**Molecular weight**	780.16 g/mol
**Chemical formula**	$C_{32}H_{14}CuNa_2O_6S_2$
**λ_{max} (nm)**	628 and 662 nm
**UV-Vis spectra at different pH**	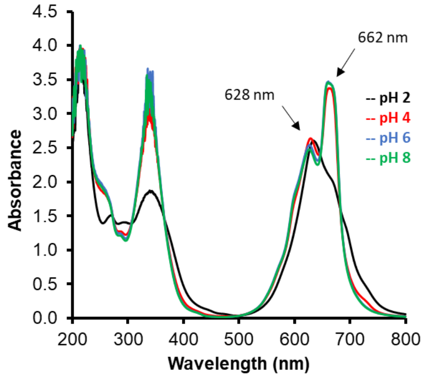

Table S2. Mathematical models used to describe the adsorption process in batch and fixed bed.

Mathematical model	Equation	Parameters
Mass Balance	$q_e = (C_o - C_e)\frac{V}{W}$	*C* C_o and C_e are the initial and equilibrium concentrations respectively (mg L^{-1}), *V* is thevolume of solution (L) and *W* is the mass of adsorbentemployed (g).
Pseudo-first order	$q_t = q_e(1 - e^{-k_1 t})$	q_e (mmol g^{-1}) and q_t (mmol g^{-1})are the amounts of adsorbed adsorbate at equilibrium and at time *t*, respectively, and k_1 (h^{-1}) is the rate constant of pseudo-first-order adsorption.
Pseudo-second order	$q_t = \frac{t}{\frac{1}{k_2 {q_e}^2} + \frac{t}{q_e}}$	k_2 (g $mmol^{-1}$ h^{-1}) is the equilibrium rate constant ofpseudo-second-order adsorption.
Elovich	$q_t = \frac{2.3}{\alpha} * \log(1 + \alpha\beta t)$	α (mmol g^{-1} min^{-1}) is the initial sorption rate, and the parameter β (g $mmol^{-1}$) is related to the extent of surface coverage and activation energy for chemisorption.

Langmuir	$q_e = \frac{q_{max} K\, C_e}{1 + KC_e}$	q_{max} is the maximum adsorption capacity of the biosorbent and *K* is the Langmuir constant indicatingan affinity for the active site.
Freundlich	$q_e = K_f {C_e}^{1/n}$	K_f is the Freundlich constantand *1/n* is the heterogeneity factor.
Temkin	$q_e = \left(\frac{RT}{b_T}\right) ln(A_T C_e)$	b_T (J/mol) is the Temkin constant related to the variation of adsorption energy, and A_T (L/mg) is the equilibrium constant of the Temkin model. *R* was the gas constant (8.314 J/mol·K), and *T* was the absolute temperature (K).
Adams-Bohart	$\frac{C}{C_0} = e^{K_{AB} C_0 t - \frac{K_{AB} N_0 Z}{v}}$	K_{AB} is the Adams-Bohart kinetic constant (L mg^{-1} min^{-1}),N_0 is the sorption capacity perunit volume of the bed (mg L^{-1}), *Z* is the length of the column bed (cm) and ν is the linear flow velocity (cm min^{-1}).
Thomas	$\frac{C}{C_0} = \frac{1}{1 + exp\left(\frac{K_{Th}}{Q}\left(q_0 m - C_0 V_{ef}\right)\right)}$	K_{Th} is the Thomas rate (mL min^{-1} mg^{-1}) and q_0 corresponds to the maximum concentration of the solute inthe solid phase (mg g^{-1}).

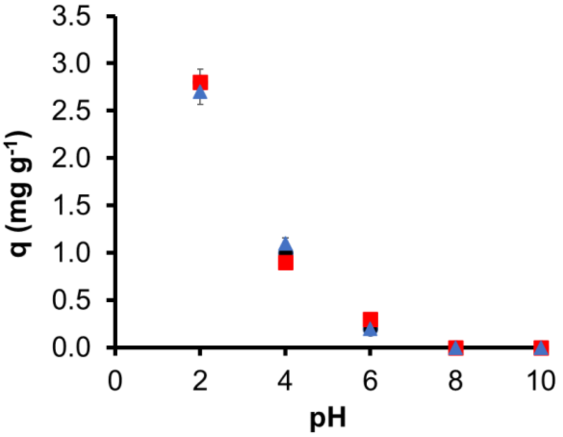

Figure S1. Effect of adsorption capacity as a function of pH in solution: ● 25 °C, ■ 35 °C, ▲ 45 °C.

Characterization of hydrochar prepared from agave bagasse

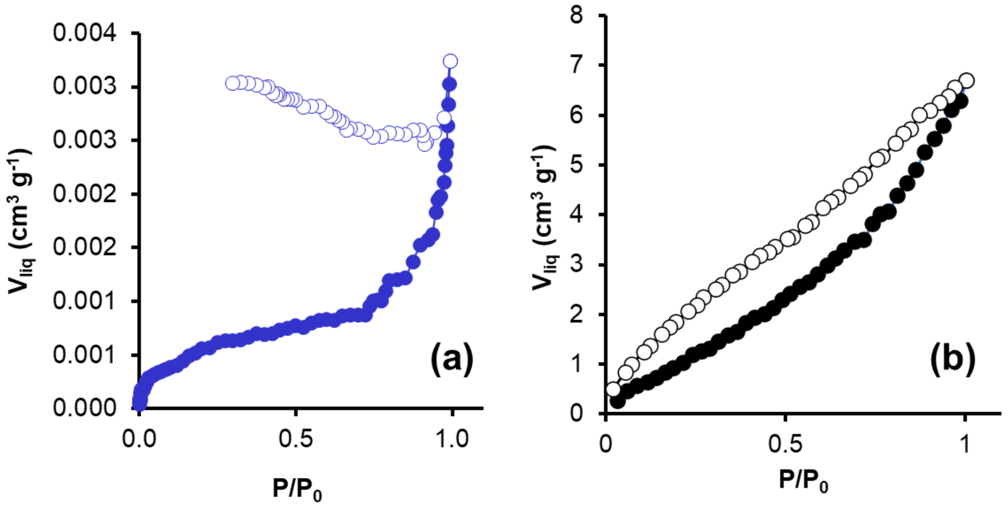

Figure S2. Nitrogen adsorption isotherms at -196 °C for: (a) biosorbent and (b) hydrochar.

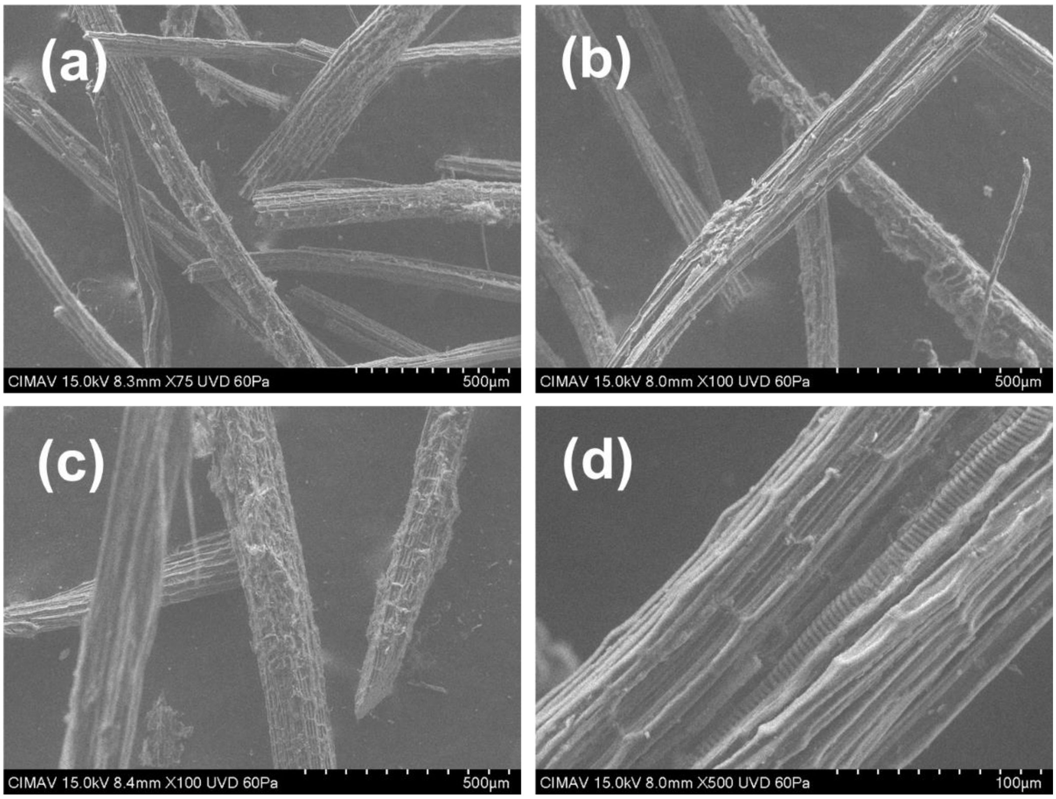

Figure S3. Microphotographs of the hydrochar prepared from agave bagasse at different magnifications.

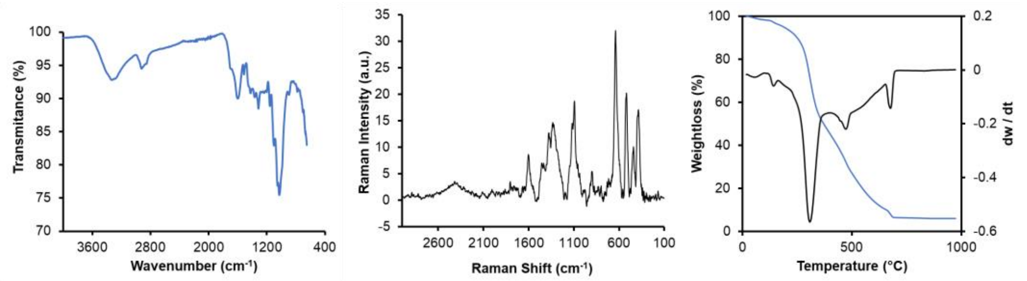

Figure S4. a) FTIR spectrum, b) RAMAN spectrum, and c) Thermogravimetric analysis of hydrochar prepared from agave bagasse.

Lyfe-cycle assessment of adsorbent materials prepared from agave bagasse

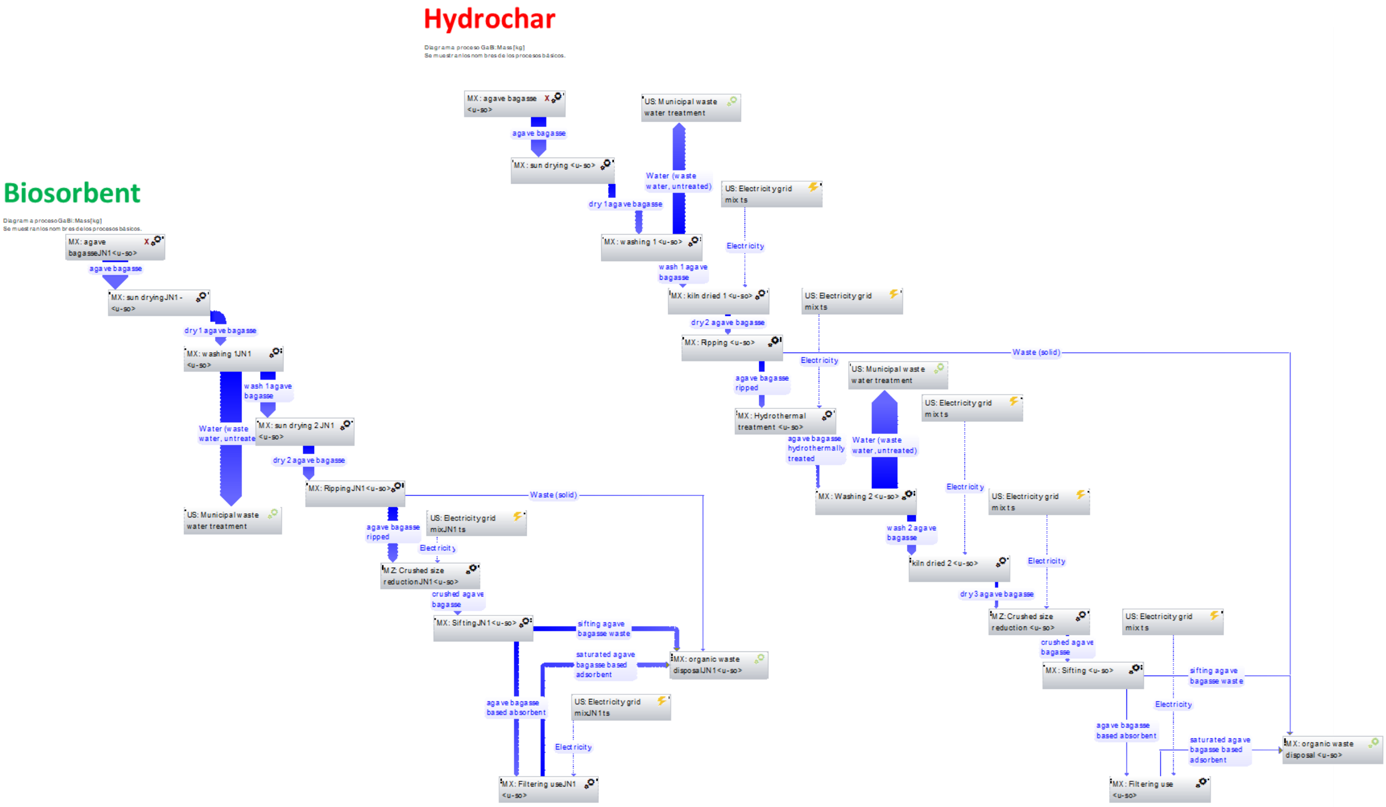

Figure S5. Diagrams created in the GaBi software for the life cycle impact assessment of both absorbents.
